# Association of Transfusion With Risks of Dementia or Alzheimer’s Disease: A Population-Based Cohort Study

**DOI:** 10.3389/fpsyt.2019.00571

**Published:** 2019-08-16

**Authors:** Shih-Yi Lin, Wu-Huei Hsu, Cheng-Chieh Lin, Cheng-Li Lin, Hung-Chieh Yeh, Chia-Hung Kao

**Affiliations:** ^1^Graduate Institute of Biomedical Sciences, College of Medicine, China Medical University, Taichung, Taiwan; ^2^Division of Nephrology and Kidney Institute, China Medical University Hospital, Taichung, Taiwan; ^3^Division of Pulmonary and Critical Care Medicine, China Medical University Hospital and China Medical University, Taichung, Taiwan; ^4^Department of Family Medicine, China Medical University Hospital, Taichung, Taiwan; ^5^Management Office for Health Data, China Medical University Hospital and Center of Augmented Intelligence in Healthcare, Taichung, Taiwan; ^6^College of Medicine, China Medical University, Taichung, Taiwan; ^7^Department of Nuclear Medicine, China Medical University Hospital, Taichung, Taiwan; ^8^Department of Bioinformatics and Medical Engineering, Asia University, Taichung, Taiwan

**Keywords:** transfusion, dementia, Alzheimer’s disease, cohort study, Taiwan National Health Insurance Research Database

## Abstract

**Purpose:** The association between neurodegenerative diseases and transfusion remains to be investigated.

**Methods:** The study population comprised 63,813 patients who underwent a blood transfusion and 63,813 propensity score-matched controls between 2000 and 2010. Data were obtained from the Taiwan National Health Insurance Research Database, which is maintained by the National Health Research Institutes. A Cox regression analysis was conducted to elucidate the relationship between blood transfusions and the risk of dementia.

**Results:** A multivariate Cox regression analysis of factors, such as age, sex, cardiovascular ischemia disease, and depression, revealed that patients who underwent a blood transfusion showed a 1.73-fold higher risk of dementia [95% confidence interval (CI) = 1.62-1.84] and a 1.37-fold higher risk of Alzheimer’s disease (AD) [95% CI = 1.13-1.66] than those who did not. Patients who received a transfusion of washed red blood cells showed a 2.37-fold higher risk of dementia (95% CI = 1.63-3.44) than those who did not.

**Conclusion:** Blood transfusion, especially transfusion of any type of red blood cells is associated with an increased risk of dementia.

## Introduction

Anemia, defined as hemoglobin concentration <12 g/dl in women and <13 g/dl in men according to World Health Organization (WHO), is a prevalent condition worldwide (1). The burden of anemia is high, an is estimated to effect between 27% to 32.9% of the world population (2, 3). Anemia is associated with increasing mortality and morbidity (4). In addition to correct underlying diseases, transfusion is a frequent clinical procedure carried out in patients with anemia (5–8). However, transfusion itself is also related to adverse events and complications (9, 10). Iron overload is one of the inevitable and notorious complications of transfusion, especially in transfusion-dependent patients (11, 12). Clinical consequences of deregulated iron metabolism and iron overload has previously been reported including hemochromatosis, insulin resistance, peripheral inflammation, cirrhosis, heart failure, and decreased survival benefits (13–17).

Recently, decreased serum iron levels have been reported to be causally associated with an increased risk of developing Parkinson’s disease (PD) (18). Abnormal deposits of iron has been observed to pathologically accumulate in the substantia nigra in PD and in the cortex in AD (19). Lei et al. have also showed that Tau deficiency could induce parkinsonism with dementia by impairing Amyloid precursor protein-mediated iron exports (20). Duce et al. have found that iron-export ferroxidase activity of the β-amyloid precursor protein undergoes interference and inhibition in AD (21). The above studies clearly demonstrate that perturbations in iron homeostasis and ferroptotic signaling are tightly associated with the development of neurodegeneration (22). Furthermore, inflammation can be induced during blood transfusion (23) and has also been reported to be involved in the pathogenesis of neurodegeneration (24).

Pathogenesis of neurodegeneration also involves misfolding protein aggregation and prorogation through anatomical connections of peripheral neurons (25, 26). Given the evidence that peripheral inoculation of the misfolded proteins can induce the aggregation of aberrant proteins in mice models and vascular inflammation is associated with dementia (27–30), the horizontal transmissibility of neurodegenerative diseases might therefore be possible (31, 32).

Edgren et al. however, reported that AD and PD cannot be transmitted through blood transfusions with blood originating from donors with misfolded proteins (33). Whether transfusion is associated with an increased risk of neurodegeneration and dementia remains unknown and called for a nationwide longitudinal cohort study. In this study, we analyzed the association between blood transfusions and the risk of dementia, using data from the National Health Insurance Research Database (NHIRD), which contains claims data of most Taiwanese citizens (> 99% coverage rate) and thus provides valuable information for epidemiological investigations.

## Methods

### Data Source

The Taiwan National Health Insurance (NHI) program was implemented in March 1995 and covers approximately 99% of Taiwanese residents (34). This retrospective population-based cohort study was conducted using data from the NHIRD for the period from 2000 to 2011. The details of the database and the program are provided in previous studies (35).

### Sampled Patients

In this study, we included patients aged ≥20 years who underwent a blood transfusion from 1 January 2000, to 31 December 2011. A blood transfusion was defined as a transfusion of packed red blood cells (RBCs), washed RBCs, frozen deglycerolized RBCs, leukocyte-poor RBCs, platelet concentrate, white blood cells (WBC) concentrate, plateletpheresis, WBCphresis, fresh frozen plasma, frozen plasma, cryoprecipitate, whole blood, and reduced leukocytes-platelets. The date of the blood transfusion was used as the index date. Patients with missing data on their date of birth and sex and those with preexisting dementia (ICD-9-CM codes 290, 331, 294.0, or 294.1) or AD (ICD-9-CM codes 290.1 and 331.0), were excluded. Further, to investigate the long-term effect of transfusion, we delayed the start of follow-ups for dementia by 2 years. Thus, persons with any dementia or AD diagnosed within 2 years after the index date were also excluded.

Every patient who underwent a blood transfusion was propensity score-matched to one randomly selected insurant without any history of blood transfusion, dementia, or AD at baseline. A logistic regression model was used to calculate propensity scores for patients in need of blood transfusion as a function of the background variables including age, sex, year of the index date, urbanization level, occupation, frequency of brain CT/MRI/per year, frequency of psychiatric outpatient visits/per year, and comorbidities. The comorbidities included in this study were diabetes (ICD-9-CM code 250), hypertension (ICD-9-CM code 401 to 405), hyperlipidemia (ICD-9-CM code 272), anxiety (ICD-9-CM code 300.00), depression (ICD-9-CM codes 296.2, 296.3, 300.4, and 311), obesity (ICD-9-CM code 278), bipolar disorder (ICD-9-CM code 296), schizophrenia (ICD-9-CM code 295), head injury (ICD-9-CM codes 800–804, 850–854, and 959.01), injury and poisoning (ICD-9-CM codes 800–999; excluding 800–804, 850–854, and 910–919), posttraumatic stress disorder (ICD-9-CM code 309.81), iron deficiency anemia (ICD-9-CM code 280.9), stroke (ICD-9-CM codes 430–438), and chronic kidney disease (CKD) and end-stage renal disease (ICD-9-CM code 585), GI bleeding (ICD-9-CM codes 530-535, 562.12, 562.13, 569.3, 569.85, 578, 455.2, 455.5, 455.8), and surgery (ICD-9-CM procedure codes 01-99). The medications included in this study were benzodiazepine (BZD), non-BZD, and antipsychotics; and the year of the index date listed in **Table 1**. The non-BZD included Zopiclone, Zolpidem hemitartrate, Zolpidem tartrate, and Zaleplon. Patients who had a blood transfusion were matched (1:1 ratio) with those who did not have a blood transfusion according to their propensity score through the nearest neighbor matching, initially to the eighth digit and then as required to the first digit. Therefore, matches were first made within a caliper width of 0.0000001, and then the caliper width was increased for unmatched cases to 0.1. We reconsidered the matching criteria and performed a rematch (greedy algorithm). The mean and median propensity scores were compared between the two cohorts.

**Table 1:** Demographic characteristics and comorbidities in cohorts with and without blood transfusion.

Variable	Blood transfusion		Standard mean difference^#^
	No	Yes	
	N = 63,813	N = 63,813	
**Age, year**			
≤49	19,470(30.5)	21,082(33.0)	0.05
50-64	19,213(30.1)	16,959(26.6)	0.08
65-79	19,927(31.2)	19,545(30.6)	0.01
80+	5,203(8.15)	6,227(9.76)	0.06
Mean ± SD	58.5 ± 16.3	58.4 ± 17.1	0.01
**Sex**			
Female	31,467(49.3)	31,199(48.9)	0.01
Male	32,346(50.7)	32,614(51.1)	0.01
**Urbanization level** ^&^			
1 (Highest urbanization)	16,305(25.6)	16,423(25.7)	0.004
2	17,853(28.0)	17,789(27.9)	0.002
3	11,361(17.8)	11,365(17.8)	0.000
4 (Lowest urbanization)	18,294(28.7)	18,236(28.6)	0.002
**Frequency of brain CT/MRI/per year, Mean ± SD**	0.28 ± 8.48	2.42 ± 31.4	0.09
**Frequency of Psychiatric OPD visit/per year, Mean ± SD**	11.6 ± 335.9	70.7 ± 1027.7	0.07
**Occupation**			
White collar	26,299(41.2)	26,071(40.9)	0.01
Blue collar	26,682(41.8)	26,798(42.0)	0.004
Others^‡^	10,832(17.0)	10,944(17.2)	0.01
**Comorbidity**			
Diabetes	10,825(17.0)	10,787(16.9)	0.002
Hypertension	29,871(46.8)	29,498(46.2)	0.01
Hyperlipidemia	16,078(25.2)	15,720(24.6)	0.01
Anxiety	5,509(8.63)	5,311(8.32)	0.01
Depression	3,725(5.84)	3,632(5.69)	0.01
Obesity	962(1.51)	944(1.48)	0.002
Bipolar disorder	375(0.59)	336(0.53)	0.01
Schizophrenia	436(0.68)	420(0.66)	0.003
Head injury	10,181(16.0)	9,840(15.4)	0.02
Injury and poisoning	29,073(45.6)	29,627(46.4)	0.02
Post-traumatic stress disorder	29(0.05)	29(0.05)	0.000
Iron deficiency anemia	3,309(5.19)	3,157(4.95)	0.01
Stroke	4,883(7.65)	5,041(7.90)	0.01
CKD and ESRD	1,631(2.56)	1,941(3.04)	0.03
GI bleeding	1,293(2.03)	1,374(2.15)	0.01
Surgery	61,269(96.0)	60,974(95.6)	0.02
**Medication**			
Benzodiazepine	23,861(37.4)	23,541(36.9)	0.01
Non-BZD	6,950(10.9)	6,838(10.7)	0.01
Anti-psychotic medications	5,208(8.16)	5,155(8.08)	0.003

^#^*A value of standard mean difference equals 0.1 or less, which indicates a negligible difference in means between blood transfusion and matched cohorts.*

^&^Urbanization level was categorized according to the population density of the residential area into four levels, with Level 1 being the most urbanized and Level 4 the least urbanized.

^‡^Other occupations included primarily retired, unemployed, or low-income populations.

### Outcome

The primary outcome of this study was a diagnosis of dementia (ICD-9-CM codes 290, 331, 294.0, or 294.1) and AD (ICD-9-CM codes 290.1 and 331.0). Each study patient was followed up with until the primary outcome was diagnosed or were censored because of death, withdrawal from the NHI program, or if after 31 December 2011.

### Statistical Analysis

The standard mean difference was used to examine the difference in categorical variables and continuous variables between the blood transfusion and matched cohorts. The standard mean difference value was 0.1 or less, which indicated a negligible mean difference between the blood transfusion and matched cohorts. The Kaplan–Meier method was used to estimate the cumulative incidence of subsequent dementia in the blood transfusion and matched cohorts, and significant differences were determined using the log-rank test. We calculated the incidence density rate of dementia according to different risk factors in analyses stratified by age, sex, urbanization level, occupation, comorbidities, and medication. Univariable and multivariable Cox proportional hazards models were used to estimate the hazard ratios (HRs) and 95% confidence intervals (CIs) to determine the association between blood transfusions and the risks of dementia or AD. The multivariable models were simultaneously adjusted for age, sex, urbanization level, occupation, frequency of brain CT/MRI/per year, and frequency of Psychiatric OPD visit/per year, and comorbidities of diabetes, hypertension, hyperlipidemia, anxiety, depression, obesity, bipolar disorder, schizophrenia, head injury, injury and poisoning, post-traumatic stress disorder, iron deficiency anemia, stroke, CKD and ESRD, GI bleeding, surgery, and medication of benzodiazepine, non-BZD, and anti-psychotic medications. A further analysis was performed to estimate the risk of dementia among patients receiving different types of blood transfusions. All statistical analyses were performed using SAS version 9.4 statistical software (SAS Institute, Inc., Cary, N.C., USA). The significance level for all analyses was set to a *p* value of 0.05.

## Results

### Demographic Characteristics, Comorbidities and Medications of Patients With Blood Transfusion and the Matched Cohort

A total of 63,813 patients were included in the blood transfusion cohort and 63,813 persons in the matched cohort, with similar distributions for age, sex, urbanization level, frequency of brain CT/MRI/per year, frequency of psychiatric outpatient visit/per year, occupation, comorbidities, and medication (**Table 1**). The median follow-up period was 3.86 ± 3.52 years in the study cohort and 5.49 ± 3.34 years in the matched cohort. In both cohorts, more than half of the patients were aged <65 years (59.6% vs 60.6%). The mean ages of the blood transfusion and matched cohorts at index date were 58.4 years (SD = 17.1) and 58.5 years (SD = 16.3), respectively. Both cohort groups mainly resided in highly urbanized areas (53.6% vs 53.6%) and were employed in blue collar jobs (42.0% vs 41.8%). The mean frequency of brain CT/MRI/per year in the matched and blood transfusion cohorts was 0.28 (SD = 8.48) and 2.42 (SD = 31.4) times per year, respectively. The major comorbidity was hypertension (46.2% vs 46.8%), followed by injury and poisoning (46.4% vs 45.6%) and hyperlipidemia (24.6% vs 25.2%). In both cohorts, the main medication used was BZD (36.9% vs 37.4%). In the category of injury and poisoning, there are 35 patients with carbon monoxide intoxication in blood transfusion cohort (0.05%; there are 29 patients with carbon monoxide intoxication in the matched cohort (0.05%)(p-value = 0.45).

### Incidence Densities and Cox Model Analysis of Risk Factors Affecting Dementia Development

The overall incidence density rates of dementia were 5.50 and 7.73 per 1000 person-years in the matched and blood transfusion cohorts, respectively (**Table 2**). Compared with the matched cohort, the adjusted HRs were 1.73 (95% CI = 1.62–1.82) for the blood transfusion cohort. Compared with individuals aged ≤49 years, the risk of dementia was 3.47-fold higher in those aged between 50 and 64 years (95% CI = 2.93–4.11), 14.5-fold higher in those aged between 65 and 79 years (95% CI = 12.4–17.0), and 38.6-fold higher in those aged ≥80 years (95% CI 32.6–45.6). Patients employed in others had a higher risk of dementia than those employed in white collar jobs. The risk of dementia was higher in patients with comorbidities including diabetes, hypertension, anxiety, depression, bipolar disorder schizophrenia, head injury, stroke, and CKD and ESRD than those who did not. Individuals using BZDs had a 1.18-fold lower risk of dementia than those who did not (95% CI = 1.10–1.27). Individuals using non-BZDs had a 1.13-fold lower risk of dementia than those who did not (95% CI = 1.02–1.25). Individuals using antipsychotics had a 1.28-fold higher risk of dementia than those who did not (95% CI = 1.16–2.42).

**Table 2:** Incidence and hazard ratio for dementia and dementia-associated risk factor.

Variable	Event	PY	Rate^#^	Crude HR(95% CI)	Adjusted HR⁑ (95% CI)
**Blood transfusion**					
No	1,926	350,237	5.50	1.00	1.00
Yes	1,905	246,438	7.73	1.50(1.41, 1.60)***	1.73(1.62, 1.84)***
**Age, year**					
≤49	191	224,930	0.85	1.00	1.00
50-64	559	173,045	3.23	4.07(3.45, 4.79)***	3.47(2.93, 4.11)***
65-79	2,280	168,216	13.6	18.4(15.9, 21.3)***	14.5(12.4, 17.0)***
80+	801	30,484	26.3	48.8(41.7, 57.2)***	38.6(32.6, 45.6)***
**Sex**					
Female	1,973	309,374	6.38	1.00	1.00
Male	1,858	287,302	6.47	1.04(0.98, 1.11)	1.05(0.99, 1.13)
**Urbanization level** ^&^					
1 (Highest urbanization)	961	153,880	6.25	1.00	1.00
2	952	168,014	5.67	0.91(0.83, 0.99)*	0.95(0.87, 1.04)
3	642	106,570	6.02	0.96(0.87, 1.06)	0.97(0.88, 1.08)
4 (Lowest urbanization)	1,276	168,211	7.59	1.22(1.13, 1.33)***	1.03(0.94, 1.14)
**Occupation**					
White collar	1,212	249,520	4.86	1.00	1.00
Blue collar	1,671	248,696	6.72	1.39(1.29, 1.50)***	0.97(0.90, 1.06)
Others^‡^	948	98,460	9.63	2.01(1.84, 2.19)***	1.14(1.04, 1.24)**
**Comorbidity**					
**Diabetes**					
No	3,023	512,955	5.89	1.00	1.00
Yes	808	83,720	9.65	1.88(1.74, 2.03)***	1.25(1.15, 1.35)***
**Hypertension**					
No	1,028	342,538	3.00	1.00	1.00
Yes	2,803	254,138	11.0	4.05(3.77, 4.35)***	1.33(1.23, 1.44)***
**Hyperlipidemia**					
No	2,522	461,375	5.47	1.00	1.00
Yes	1,309	135,300	9.67	1.95(1.83, 2.09)***	0.98(0.91, 1.05)
**Anxiety**					
No	3,378	555,991	6.08	1.00	1.00
Yes	453	40,685	11.1	2.22(2.01, 2.45)***	1.17(1.05, 1.30)**
**Depression**					
No	3,432	567,118	6.05	1.00	1.00
Yes	399	29,557	13.5	2.52(2.27, 2.80)***	1.61(1.44, 1.82)***
**Obesity**					
No	3,785	589,077	6.43	1.00	1.00
Yes	46	7,598	6.05	1.09(0.81, 1.45)	1.19(0.89, 1.59)
**Bipolar disorder**					
No	3,800	593,855	6.40	1.00	1.00
Yes	31	2,820	11.0	1.94(1.36, 2.76)***	1.64(1.14, 2.36)**
**Schizophrenia**					
No	3,806	593,039	6.42	1.00	1.00
Yes	25	3,636	6.88	1.15(0.77, 1.70)	2.40(1.60, 3.59)***
**Head injury**					
No	3,235	517,677	6.25	1.00	1.00
Yes	596	78,998	7.54	1.36(1.25, 1.49)***	1.42(1.29, 1.55)***
**Injury and poisoning**					
No	2,502	342,515	7.30	1.00	1.00
Yes	1,329	254,161	5.23	0.76(0.71, 0.81)***	0.83(0.77, 0.89)
**Post-traumatic stress disorder**					
No	3,829	596,483	6.42	1.00	1.00
Yes	2	193	10.4	2.16(0.54, 8.64)	2.36(0.59, 9.46)
**Iron deficiency anemia**					
No	3,721	568,758	6.54	1.00	1.00
Yes	110	27,917	3.94	0.66(0.54, 0.80)***	1.14(0.94, 1.38)
**Stroke**					
No	3,294	563,126	5.85	1.00	1.00
Yes	537	33,550	16.0	3.28(2.99, 3.59)***	1.87(1.70, 2.05)***
**CKD and ESRD**					
No	3,713	582,438	6.37	1.00	1.00
Yes	118	14,237	8.29	1.45(1.21, 1.74)***	1.37(1.14,1.65)***
**GI bleeding**					
No	3,775	585,860	6.44	1.00	1.00
Yes	56	10,815	5.18	0.92(0.71, 1.20)	1.02(0.78, 1.33)
**Surgery**					
No	190	31,406	6.05	1.00	1.00
Yes	3,641	565,269	6.44	1.21(1.05, 1.41)**	1.03(0.89, 1.19)
**Medication**					
**Benzodiazepine**					
No	1,814	396,973	4.57	1.00	1.00
Yes	2,017	199,702	10.1	2.46(2.31, 2.62)***	1.18(1.10, 1.27)***
**Non-BZD**					
No	3,277	548,529	5.97	1.00	1.00
Yes	554	48,147	11.5	2.42(2.21, 2.64)***	1.13(1.02, 1.25)*
**Anti-psychotic medications**					
No	3,307	555,310	5.96	1.00	1.00
Yes	524	41,365	12.7	2.38(2.17, 2.61)***	1.28(1.16, 1.42)***

Rate^#^, incidence rate, per 1,000 person-years; Crude HR, relative hazard ratio; Adjusted HR^⁑^, adjusted for age, sex, urbanization level, occupation, frequency of brain CT/MRI/per year, and frequency of Psychiatric OPD visit/per year, and comorbidities of diabetes, hypertension, hyperlipidemia, anxiety, depression, obesity, bipolar disorder, schizophrenia, head injury, injury and poisoning, post-traumatic stress disorder, iron deficiency anemia, stroke, CKD and ESRD, GI bleeding, surgery, and medication of benzodiazepine, non-BZD, and anti-psychotic medications.

*^&^*Urbanization level was categorized according to the population density of the residential area into four levels, with Level 1 being the most urbanized and Level 4 the least urbanized.

^‡^Other occupations included primarily retired, unemployed, or low-income populations.

*p < 0.05, **p < 0.01, ***p < 0.001.

### Cox Model With Hazard Ratios and 95% Confidence Intervals of Alzheimer’s Disease Between the Blood Transfusion and Matched Cohorts

The overall incidence density rates of AD were 0.68 and 0.77 per 1000 person-years in the matched and blood transfusion cohorts, respectively (**Table 3**). Compared with the matched cohort, the HR of AD was 1.37 (95% CI = 1.13–1.66) in the blood transfusion cohort.

**Table 3:** Hazard ratio (HR) of Alzheimer’s disease for the blood transfusion cohort relative to the non-blood transfusion cohort.

	Blood transfusion	
	No	Yes
	N = 63,813	N = 63,813
**Alzheimer’s disease**		
Event	240	192
Person-years	352,911	249,158
Rate ^#^	0.68	0.77
**Crude HR(95% CI)**	1(Reference)	1.20(1.00, 1.46)*
**Adjusted HR (95% CI)** **^⁑^**	1(Reference)	1.37(1.13, 1.66)**

*Rate* *^#^* *, incidence rate, per 1,000 person-years; Crude HR, relative hazard*

ratio; Adjusted HR^⁑^, adjusted for age, sex, urbanization level, occupation, frequency of brain CT/MRI/per year, and frequency of Psychiatric OPD visit/per year, and comorbidities of diabetes, hypertension, hyperlipidemia, anxiety, depression, obesity, bipolar disorder, schizophrenia, head injury, injury and poisoning, post-traumatic stress disorder, iron deficiency anemia, stroke, CKD and ESRD, GI bleeding, surgery, and medication of benzodiazepine, non-BZD, and anti-psychotic medications.

*p < 0.05; **p < 0.01.

### Incidence and HRs of Dementia Comparison Different Type Blood Transfusion and Matched Cohorts

Additionally, we analyzed the incidence and HR of dementia according to the different types of blood transfusions (**Table 4**). Patients who underwent packed RBCs transfusion, washed RBCs, Leukocyte-poor RBCs, platelet concentrate or FFP had a significantly higher risk of dementia than the matches.

**Table 4:** Incidence and hazard ratio of dementia between different type blood transfusions.

Variables	N	Event	Rate^#^	Crude HR(95% CI)	Adjusted HR^⁑^ (95% CI)
**Blood transfusion**					
Without blood transfusion	63,818	1,926	5.50	1(Reference)	1(Reference)
Packed RBCs	57,785	1,779	7.90	1.53(1.44, 1.64)***	1.80(1.69,1.92)***
Washed RBCs	568	28	11.7	2.29(1.57, 3.32)***	2.37(1.63, 3.44)***
Frozen deglycerolized RBCs	1	0	0.00	–	–
Leukocyte-poor RBCs	998	15	5.17	1.21(0.73, 2.01)	1.72(1.03, 2.86)*
Platelet concentrate	855	21	6.32	1.21(0.79, 1.86)	2.04(1.32, 3.13)**
Leukocyte concentrate	0	0	0.00	–	–
Plateletpheresis	439	5	4.18	0.87(0.36, 2.09)	1.14(0.47, 2.73)
Leukocytaphresis	25	0	0.00	–	–
Fresh frozen plasma	2,482	45	6.13	1.18(0.88, 1.59)	1.55(1.15, 2.08)**
Frozen plasma	366	3	4.81	1.04(0.34, 3.24)	1.66(0.54, 5.17)
Cryoprecipitate	26	0	0.00	–	–
Whole blood	442	9	2.97	0.46(0.24, 0.88)*	0.93(0.48, 1.80)
RL-platelets	21	0	0.00	–	–

Rate^#^, incidence rate, per 1,000 person-years; Crude HR, relative hazard ratio; Adjusted HR^⁑^, adjusted for age, sex, urbanization level, occupation, frequency of brain CT/MRI/per year, and frequency of Psychiatric OPD visit/per year, and comorbidities of diabetes, hypertension, hyperlipidemia, anxiety, depression, obesity, bipolar disorder, schizophrenia, head injury, injury and poisoning, post-traumatic stress disorder, iron deficiency anemia, stroke, CKD and ESRD, GI bleeding, surgery, and medication of benzodiazepine, non-BZD, and anti-psychotic medications; RBC, red blood cells; RL, reduced leukocytes.

*p < 0.05, **p < 0.01, ***p < 0.001.

### Cumulative Incidence of Dementia and Dementia Event Risk According to Follow-Up Years in the Blood Transfusion and Matched Cohorts

**Figure 1:**
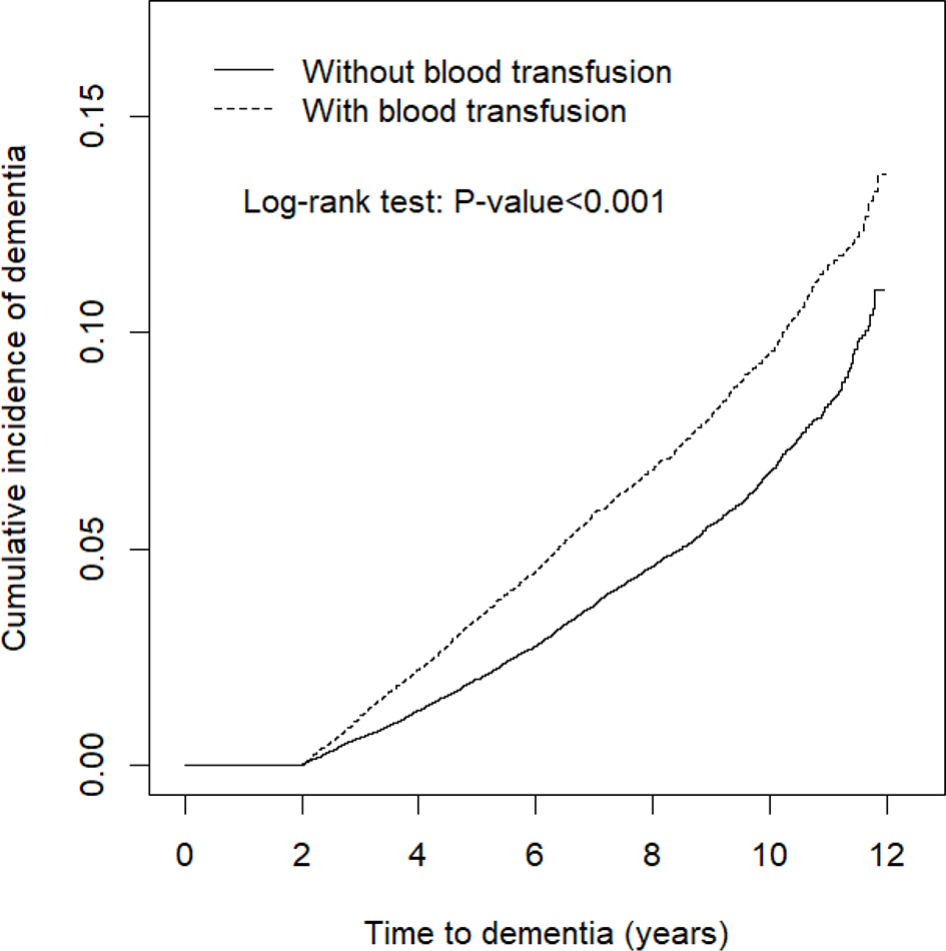
Cumulative incidence comparison of dementia for patients with (dashed line) or without (solid line) blood transfusion.

The results indicated that at the end of the follow-up period, the cumulative incidence of dementia was higher in the blood transfusion cohort than in the matched cohort (log-rank test, *p* < 0.001) (**Figure 1**). The risk of dementia fell over time but persisted through the follow-up period (**Table 5**). The higher risk occurred during the first 3 y of the follow-up period (adjusted HR = 2.02, 95% CI = 1.74–2.35), and reduced with increasingly longer periods to 1.87 between 3 to 6 y of follow-ups. The risk remained for 6 y of follow-ups.

**Table 5:** Incidence of dementia by follow-up period and Cox model measured hazards ratio for patients with blood transfusions compared those without blood transfusions.

Variables	Blood transfusion						Crude HR (95% CI)	Adjusted HR^⁑^ (95% CI)
	No			Yes				
	Event	PY	Rate^#^	Event	PY	Rate^#^		
**Follow-up period**								
≤3	309	163,966	1.88	382	125,013	3.06	1.75(1.51, 2.03)***	2.02(1.74, 2.35)***
3-6	777	109,755	7.08	834	72,664	11.5	1.62(1.47, 1.79)***	1.87(1.69, 2.06)***
≥6	840	76,515	11.0	689	48,761	14.1	1.29(1.16, 1.42)***	1.56(1.41, 1.73)***

PY, person-years; Rate^#^, incidence rate, per 1,000 person-years; Crude HR, relative hazard ratio; Adjusted HR^⁑^, adjusted for age, sex, urbanization level, occupation, frequency of brain CT/MRI/per year, and frequency of Psychiatric OPD visit/per year, and comorbidities of diabetes, hypertension, hyperlipidemia, anxiety, depression, obesity, bipolar disorder, schizophrenia, head injury, injury and poisoning, post-traumatic stress disorder, iron deficiency anemia, stroke, CKD and ESRD, GI bleeding, surgery, and medication of benzodiazepine, non-BZD, and anti-psychotic medications. ***p < 0.001.

### Relationship Between the Frequency of Blood Transfusion and the Risk of Dementia Development

The risk of dementia development was higher in patients presenting with 1, 2 or ≥3 blood transfusions compared with those in the matched cohort (p for trend <0.001) (**Table 6**).

**Table 6:** The incidence rate and risk of dementia in patients with blood transfusions, stratified by frequency of blood transfusions.

Blood transfusion	Event	Person-years	Rate^#^	Adjusted HR (95% CI)^⁑^	Adjusted HR (95% CI)^⁑^
**All**					
0	1,926	350,237	5.50	1.00	
1	702	108,267	6.48	1.86(1.71, 2.03)***	1.00
2	413	47,455	8.70	1.85(1.67, 2.06)***	1.01(0.90, 1.15)
≥3	790	90,716	8.71	1.69(1.56, 2.06)***	0.93(0.84, 1.03)
P for trend				< 0.001	0.16

Rate^#^, incidence rate, per 1,000 person-years; Adjusted HR^⁑^, adjusted for age, sex, urbanization level, occupation, frequency of brain CT/MRI/per year, and frequency of Psychiatric OPD visit/per year, and comorbidities of diabetes, hypertension, hyperlipidemia, anxiety, depression, obesity, bipolar disorder, schizophrenia, head injury, injury and poisoning, post-traumatic stress disorder, iron deficiency anemia, stroke, CKD and ESRD, GI bleeding, surgery, and medication of benzodiazepine, non-BZD, and anti-psychotic medications. ratio; ***p < 0.001.

## Discussion

This study showed an association between blood transfusions and the risk of dementia. There are several possible explanations for our findings. First, transfusion might cause dysregulated iron metabolism in transfused patients. Lei et al. have also showed that Tau deficiency could induce parkinsonism with dementia by impairing the Amyloid precursor protein-mediated iron export (20). Duce et al. have found that iron-export ferroxidase activity of the β-amyloid precursor protein undergoes interference and is inhibited in AD (21). Transfusion has been linked with dysregulated iron metabolism in chronic transfused patients (14) and iron has been implicated in the pathology of many neurodegenerative diseases (36–39). Therefore, the dysregulated metabolism of iron as a result of transfusion might assist with the development of dementia in transfused patients. Second, transfusion-related inflammation might be involved in the pathogenesis of dementia. Patients who require transfusion are those with many comorbidities including diabetes mellitus, hypertension, chronic kidney disease, and stroke etc. It has been reported that transfusion could evoke inflammation and associated inflammatory cytokines. Cognasse et al. stated that microparticles originating from platelets, leukocytes, erythrocytes, and endothelial cells can trigger a pro-inflammatory message during transfusion (40). Chronic inflammation has been linked to neurodegenerative changes in the brain and the risks of dementia (41–44). Engelhart et al. also reported that increased levels of the plasma protein are associated with risks of dementia (45). Therefore, acute peripheral inflammation evoked during transfusion and chronic peripheral inflammation as a result of underlying comorbidities might both contribute to the increasing risk of dementia in transfused patients. Third, microparticles, e.g., exosomes or red blood cells in blood might play a role as carriers for molecules that may be related to the risk of dementia. Recent studies have demonstrated that neural-derived blood exosomes could predict or identify the development of dementia (46–49). Kapoginnias et al. identified that dysfunctional phosphorylated type 1 insulin receptor substrate in neural-derived blood exosomes could predict the development of dementia (48). This proposed mechanism might account for our findings that transfusion of different prepared blood products is associated with different risks of dementia. Transfusion of all types of RBC, platelet concentrate, and fresh frozen plasma were especially at risk of dementia. Further, Rieux et al. and Bu et al. have found that mutant huntingtin could be transferred *via* the bloodstream, cross the blood-brain-barrier and enter the brain parenchyma of mice (50, 51). However, since information about dementia of donors was unavailable, our study could not establish a direct relationship that neurodegenerative disease could be transmitted *via* blood transfusion. The mechanism remains unknown and future laboratory studied are needed to clarify the pathway.

Although both our study and the Edgren et al. study (33) investigated whether transfusion was associated with the risk of neurodegenerative disease, the two studies are not really comparable because the study designs were different. Several distinct differences in the present study and the Edgren et al. study should be mentioned here for future considerations and studies (33). First, Edgren et al. (33) reported that the risk of neurodegenerative diseases in recipients of blood transfusions from donors with a record of neurodegenerative disease was insignificant, while our study was an external comparison of the risk of neurodegenerative disease between recipients of blood from unknown donors and persons with no history of blood transfusion. Second, we have matched considerable dementia- associated comorbidities and medications, while Edgren et al. (33) considered age, gender, country, and diseases of donors. Chasse et al. investigated the association between blood donor age and sex with recipient survival (52). Chasse et al. considered many confounders in their study (52). From the study design of Chaase et al. (52), it is reasonable to surmise that confounders are critically important when investigating the consequences of blood transfusion.

Our data also showed that the risk of dementia was significantly higher, especially within 3 years following blood transfusion. Patients who require blood transfusion require more medical visits following the blood transfusion, thus providing more chances of finding dementia. Another supposed reason would be the competing risk that patients who require blood transfusions might have a shortened survival duration; thus, the risk of dementia seems decreased with years following transfusion.

Several limitations of this study should be mentioned. First, information on the apolipoprotein E genotype and the family history of each individual is unavailable. The influence of genetics could therefore not be evaluated in this study. Second, data on homocysteine levels (30), cholesterol levels, blood glucose levels, blood pressure, body mass index, smoking habits, alcohol consumption, sun exposure, and education levels, which are potential confounding factors of dementia, are unavailable in the NHIRD. However, to minimize the possible bias, we matched sex; occupation; associated comorbidities, including obesity, hypertension, diabetes, hyperlipidemia, and CKD (which is associated with a high homocysteine level); and medication associated with dementia. It should be noted that although we have utilized occupation as a proxy for educational level, occupation is not directly tightly associated with education level. Therefore, our study results showed that those with white collar jobs had a lower risk of dementia, which could not absolutely be caused by the higher level of education. Third, this study was conducted with registry data while dementia is often missed in registry data. Therefore, the events and risks of dementia might be underestimated. Fourth, the possibility of confounding through the indication of blood transfusion should also be considered. We have added gastrointestinal bleeding and surgery as the two circumstances that most warrant transfusion as variables to lessen the possible baseline bias. Fifth, information of variables including APOE, hippocampal volume or global atrophy or amnestic phenotype was unavailable in this study, which could potentially cause bias. Sixth, this is an external study and since those who need a transfusion might not be as likely as those who did not need a transfusion, a potential baseline bias could exist. However, we have done propensity matching to minimize this bias. Finally, whether blood donors had neurodegenerative diseases was unknown in this study. The medical follow up was short, which might not allow for enough time to assess for the incidence of AD or dementia cases in the young patient category. Further, each individual has a different post-transduction period and the number of doctor visits, in order to monitor his/her health status, was another limitation. The direct relationship between neurodegenerative diseases and blood transfusions could not be established in this study.

In conclusion, the results of the current study indicate that blood transfusions are associated with risks of dementia or AD. Transfusion is associated with the risk of dementia, regardless of transfusion frequency. Additional research is required to elucidate the biological mechanism underlying this association. This study promotes clinical alertness relating to the safety of blood transfusions, beyond previously known issues of hepatitis and HIV.

## Data Availability

The dataset used in this study is held by the Taiwan Ministry of Health and Welfare (MOHW). The Ministry of Health and Welfare must approve the application to access this data. Any researcher interested in accessing this dataset can submit an application form to the Ministry of Health and Welfare requesting access and contact the staff of MOHW (Email: stcarolwu@mohw.gov.tw) for further assistance. Taiwan Ministry of Health and Welfare Address: No.488, Sec. 6, Zhongxiao E. Rd., Nangang Dist., Taipei City 115, Taiwan (R.O.C.). Phone: +886-2-8590-6848. All relevant data are within the paper.

## Ethics Statement

The NHIRD encrypts patients’ personal information to protect privacy and provide researchers with anonymous identification numbers associated with relevant claims information, including sex, date of birth, medical services received, and prescriptions. Therefore, patient consent is not required to access the NHIRD. This study was approved to fulfill the condition for exemption by the Institutional Review Board (IRB) of China Medical University (CMUH-104-REC2-115-CR3). The IRB also specifically waived the consent requirement.

## Author Contributions

S-YL and C-HK contributed to the conceptualization. Methodology, software, , and resources were handled by C-LL and C-HK. Validation, formal analysis, data curation, original draft preparation, review and editing, and visualization were handled by S-YL, C-LL, C-CL, H-CY, W-HH, and C-HK. Supervision, project administration, and funding acquisition were handled by C-HK.

## Funding

This work was supported by grants from the Ministry of Health and Welfare, Taiwan (MOHW108-TDU-B-212-133004), the China Medical University Hospital (DMR-107-192); the Academia Sinica Stroke Biosignature Project (BM10701010021); the MOST Clinical Trial Consortium for Stroke (MOST 107-2321-B-039 -004-); the Tseng-Lien Lin Foundation, Taichung, Taiwan; and the Katsuzo and Kiyo Aoshima Memorial Funds, Japan. The funders had no role in study design, data collection and analysis, decision to publish, or preparation of the manuscript. No additional external funding was received for this study.

## Conflict of Interest Statement

The authors declare that the research was conducted in the absence of any commercial or financial relationships that could be construed as a potential conflict of interest.

## Abbreviations

CI, confidence interval; NHIRD, National Health Insurance Research Database; ICD-9-CM, International Classification of Diseases, Ninth Revision, Clinical Modification.
